# DASH, the data and specimen hub of the National Institute of Child Health and Human Development

**DOI:** 10.1038/sdata.2018.46

**Published:** 2018-03-20

**Authors:** Rohan Hazra, Susan Tenney, Alexandra Shlionskaya, Rajni Samavedam, Kristin Baxter, John Ilekis, Jennifer Weck, Marian Willinger, Gilman Grave, Katerina Tsilou, David Songco

**Affiliations:** 1 Eunice Kennedy Shriver National Institute of Child Health and Human Development, Maternal and Pediatric Infectious Disease Branch, Bethesda, Maryland 20817, USA; 2 Booz Allen Hamilton, Inc., McLean, Virginia 22102, USA; 3 Eunice Kennedy Shriver National Institute of Child Health and Human Development, Pregnancy and Perinatology Branch, Bethesda, Maryland 20817, USA; 4 Eunice Kennedy Shriver National Institute of Child Health and Human Development, Division of Intramural Population Health Research, Bethesda, Maryland 20817, USA; 5 Eunice Kennedy Shriver National Institute of Child Health and Human Development, Pediatric Growth and Nutrition Branch, Bethesda, Maryland 20817, USA; 6 Eunice Kennedy Shriver National Institute of Child Health and Human Development, Obstetric and Pediatric Pharmacology and Therapeutics Branch, Bethesda, Maryland 20817, USA; 7 Eunice Kennedy Shriver National Institute of Child Health and Human Development, Information Resources Management Branch, Bethesda, Maryland 20817, USA

**Keywords:** Databases, Translational research

## Abstract

The benefits of data sharing are well-established and an increasing number of policies require that data be shared upon publication of the main study findings. As data sharing becomes the new norm, there is a heightened need for additional resources to drive efficient data reuse. This article describes the development and implementation of the Data and Specimen Hub (DASH) by the **Eunice Kennedy Shriver** National Institute of Child Health and Human Development (NICHD) to promote data sharing from NICHD-funded studies and enable researchers to comply with NIH data sharing policies. DASH’s flexible architecture is designed to archive diverse data types and formats from NICHD’s broad scientific portfolio in a manner that promotes FAIR data sharing principles. Performance of DASH over two years since launch is promising: the number of available studies and data requests are growing; three manuscripts have been published from data reanalysis, all within two years of access. Critical success factors included NICHD leadership commitment, stakeholder engagement and close coordination between the governance body and technical team.

## Introduction

The benefits of data sharing on public health outcomes are well-established. Several major life-saving and scientific breakthroughs were made possible through data sharing – the use of aspirin to reduce deaths from heart attacks as well as recent breakthroughs in schizophrenia, cancer and other genetic diseases^1–4^. A growing body of evidence shows that when data are made accessible, they are frequently reused by other researchers to the benefit of all. For example, two recent studies found that between 15 and 20 percent of datasets archived in a public repository are reused^5,6^ and that publications where the data were made available through a public repository received more citations than similar studies where the data were not made available^6,7^. A recent analysis of the reuse of clinical trial data made available through a centralized data repository highlights that data sharing can benefit the scientific community in a variety of ways including testing new research questions or hypotheses, testing statistical methods, performing meta-analyses, and developing predictive algorithms^8^.

In recognition of the benefits of data sharing, many funding agencies and governance bodies have issued policies requiring research data be made available upon study completion. The National Institutes of Health (NIH) has issued policies mandating timely sharing of research data by investigators receiving more than $500,000 in direct costs from NIH in any one year^9^. A similar policy was later enacted to apply to all NIH-funded genome-wide association studies^10^ and intramural research^11^. In 2012, the trans-NIH Big Data to Knowledge (BD2K) initiative was launched to facilitate the reuse of biomedical data by improving the accessibility of data and developing tools and software to aid in data analysis^12^. More recently, passage of the 21^st^ Century Cures Act^13^ has further strengthened NIH’s ability to require data sharing as a condition of funding. Beyond NIH, the International Committee of Medical Journal Editors (ICMJE) will enact a policy in 2018 that requires manuscripts submitted to ICMJE journals that report the results of a clinical trial to include a data sharing statement^14^. For clinical trials that begin enrolling participants on or after January 1, 2019, the ICMJE policy will require that a data sharing plan is included in the trial’s registration as a condition for publication in an ICMJE journal^14^.

As data sharing becomes the new norm, there is heightened awareness of the need for additional resources and infrastructure to facilitate the meaningful reuse of biomedical data. There is growing recognition that resources used in the past, such as institute-level webservers and online supplementary data links associated with publications are not well suited to the task of ensuring data are available and can be meaningfully reused by the broader community^15,16^. In recognition of this unmet need, guidelines for increasing the findability, accessibility, interoperability and reusability (FAIR) of datasets were recently published^17^ and have been embraced by many important funding agencies, including NIH. For example, under the BD2K program, NIH has recently launched a Data Commons Pilot Phase^18^ that will test ways to store, access and share biomedical data and associated tools in accordance with FAIR principles.

Centralized data repositories play a critical role in addressing many of the challenges associated with data sharing. Centralized repositories can facilitate data sharing by breaking down the silos that have traditionally made data more difficult to locate and access. However, simply cataloging existing data does not drive meaningful reuse of data. For centralized repositories to enhance the reusability of existing biomedical datasets, they must embrace FAIR principles. Examples of how centralized repositories can embrace FAIR principles include the use of metadata tags to improve the findability of datasets and ensure that important study documents and objects, including the protocols, tools, algorithms, and terminology standards used to generate the data are available to researchers to aid in the re-analysis and interpretation of the data.

In light of the critical role that centralized repositories play in facilitating data sharing, the **Eunice Kennedy Shriver** National Institute of Child Health and Human Development (NICHD) has established a centralized repository - the Data and Specimen Hub (DASH) - to enable sharing and meaningful re-use of data from the intramural and extramural clinical research projects funded through NICHD. NICHD is one of the 27 Institutes and Centers at NIH with a $1.38 billion congressional appropriation in fiscal year 2017. Founded in 1962, NICHD’s mission is to ensure that every person is born healthy and wanted, that women suffer no harmful effects from reproductive processes, and that all children have the chance to achieve their full potential for healthy and productive lives. This paper describes the rationale for establishing DASH to accommodate NICHD’s diverse study portfolio and how DASH uses FAIR principles to address many of the challenges that have historically limited data sharing.

## Results

### Decision to Establish a Data Repository

The need for a central data repository was initially identified by the broader scientific community during the NICHD’s scientific visioning process in 2011. The visioning process engaged over 700 multidisciplinary experts from the child health and human development research community with the goal of establishing shared views of where and how to direct NICHD funded research in the upcoming decade. One of the common themes that emerged was the need to find more efficient ways to store, share and analyze data^19^. In establishing DASH, a centralized data repository for NICHD-funded clinical research studies, NICHD’s goal is to promote new research and the testing of new or alternative hypotheses and analytic methods. Doing so encourages a diversity of analyses and opinions and facilitates the education of new researchers. A centralized repository enables the exploration of topics not originally envisioned by the initial investigator, reinforcing open scientific inquiry. It also provides an avenue for independent verification of research data and analyses, in keeping with broader initiatives underway at NIH to enhance rigor and reproducibility in research^20^.

### Leverage an existing repository or build something new?

After committing to a centralized clinical data repository, NICHD Leadership established the DASH Committee to oversee its planning, development and maintenance. To determine whether to leverage an existing data repository or build an entirely new system, the Committee undertook an environmental scan to determine if there was an existing NIH data repository that could accommodate the breadth and depth of clinical research studies funded through NICHD. The Committee’s decision to leverage an existing system or build a new one was influenced by several considerations. Foremost was the uniqueness and diversity of studies in the NICHD research portfolio. NICHD with its broad mission supports research on population groups that spans from pre-conception to old age across U.S. and international locations. This generates a wealth of data that vary greatly in size, structure and topics (ranging from developmental disorders such as autism to infectious diseases such as HIV/AIDS), and from single sites with less than a hundred participants to multiple sites enrolling thousands. A scan of the types and size of data that needed to be archived revealed a diversity of data types including audio files and images. The data ranged in size from 15 MB to more than 20 TB. A second determining factor for the Committee was the study specific governance that is required during data submission and request to ensure that DASH meets the needs of the diverse portfolio of studies funded by NICHD.

The environmental scan of existing NIH data repositories identified several important gaps from the perspective of the NICHD research community. Notably, many of the existing repositories had restrictions around subject matter and the type of data or metadata accepted into the repository that would exclude most clinical research data in need of archiving from NICHD-funded studies. The repositories surveyed also had limited data search capabilities, particularly semantic searches and searches for study documents and objects. The environmental scan also revealed a dearth of data visualization tools, limited adoption of terminology standards, minimal flexibility in the systems architecture and limited use of cloud technology. For these reasons, the DASH Committee decided that the best course of action would be to embark on building DASH - a new publicly accessible data and specimen repository designed to meet the needs of the NICHD clinical research community.

A key aspect to the Committee’s planning of DASH was to develop specific goals for the repository, which then dictated the design requirements and ultimately the success criteria based on which the performance and return on investment (ROI) of the repository would be evaluated (Table 1). The ROI evaluation is intended to capture the ‘return’ in the broadest possible sense including the scientific impact of the findings resulting from data reuse as well as the financial and human resource efficiencies inherent in reusing existing data rather than generating new datasets. The evaluation of ROI will include metrics such as the number of publications resulting from secondary reuse of data obtained from DASH, the impact factor of the journal in which the secondary reuse studies are published, the significance of the impact of findings resulting from secondary data reuse through the H-index and the number of new collaborations initiated from sharing data in DASH.

### How DASH addressed common data sharing challenges and made the data FAIR

Launched in the Fall of 2015, DASH addresses many of the challenges that have historically limited data sharing (Table 2). DASH provides a platform to share data from completed studies and is ready to accept data as soon as the data are ready for dissemination. In doing so, it provides investigators with another mechanism to meet the data sharing requirements laid out in the 2003 NIH Data Sharing Policy^1^.

DASH has also helped modernize how data are shared - by storing data that is FAIR^16^. In accordance with FAIR principles, all studies archived in DASH are annotated with standard metadata tags to maximize their *findability*. DASH is metadata driven – that is, the metadata for each dataset or document is dynamically driven in the system by data submitter entries and automatically populates the different search facets/filters of the search page. Users can locate studies and study files archived in DASH using a keyword semantic search or through the study catalog which allows users to browse by topic, study type or life stage. The search page has additional filters including data and document types to assist users to find what they need with fewer clicks. Proper annotations of the data and documents is critical to efficient searches and discoverability of files stored in DASH, which in turn promotes data reuse. To ensure that all study files (data and documents) are appropriately annotated before archiving and to make the task convenient for submitting investigators, DASH provides an offline and downloadable Data Preparation Tool to facilitate cataloging and annotating the study files, and prepare the study schema prior to uploading the data. To keep the annotation consistent across all studies in DASH, certain fields in the Data Preparation Tool are mandated. Each study archived in DASH has a study overview page with a brief description of the study along with information on the study timeline, population, schema, and documentations. The study overview page also includes links to related studies, such as precursor studies or sub-studies, with available datasets archived in DASH or another external repository.

DASH is designed to ensure that data are easily *accessible* by making the process for obtaining data clear, fair and transparent. The study overview page helps ensure that the data and documentations are not only findable but also accessible – for example, the study overview page provides easy access to search study data (listing each file with a brief description) and download all associated documentations such as the study protocol, data collection instruments, data dictionary, and data de-identification methodology. All users can browse studies and search for data available through DASH. Registered users can download documentation and request data. Access to data requires approval from DASH Data Access Committee (and in some cases, by a study-specific entity such as the Study Steering Committee) and execution of the DASH Data Use Agreement^21^ by the requesting investigator’s institution and NICHD. As of February 2018, DASH has received 73 data access requests, all of which were approved and resulted in access to the data. The average turnaround time from when the data request is submitted to DASH to approval of it has been seven days with no requests taking more than three weeks (21 days) to process. Maintaining a process for data access that is transparent, fair and timely is essential for the continued adoption and growth of DASH.

To ensure *interoperability,* DASH stores all data as “digital objects” and assigns each object a unique identifier at the time of submission. This identifier is retained when the object is requested by a user. It can also be used for interoperability with another data archive system. During study submission process, digital objects are grouped based on their type – datasets, documents, images. Each object is annotated with structured, standardized metadata attributes and the set of attributes depends on the type of object. When possible, metadata values for annotation are derived from established standardized resources, such as CDISC and NCI Thesaurus, and presented to the User as a code list to select during annotation process.

DASH has established mandatory requirements to help ensure that the data stored in DASH are *reusable* and can be assessed for their quality. DASH only houses studies with accompanying essential documentation, including the protocol, data collection instruments, data dictionary or codebook, and data de-identification methodology that can adequately describe data. For older studies with limited resources to prepare data for sharing, DASH staff expertise is available to investigators to generate codebooks with frequency statistics as well as data workflows and explanatory documents. In addition to the mandatory documentation, data submitters are also encouraged to include other important study documents and objects that will aid in the understanding and interpretation of that data such as the data collection methodology and data analysis plan.

DASH also provides assurances for data privacy and security. First, DASH requires that data submitted for archiving be deidentified of all 18 HIPAA identifiers and only disseminates such deidentified data. Although responsibility for de-identification resides with the study investigator, DASH offers various resources to assist investigators, including guidance documents and data curators to verify data de-identification. Second, DASH requires institutional signoff from submitting and requesting institutions to ensure that the burden of protecting privacy and confidentiality is shared between NICHD and the submitting or requesting institutions. For data submissions, DASH requires an institutional certification whereby the submitting institution certifies that the data has been de-identified and an IRB or an equivalent Privacy Board has reviewed and verified that the data being submitted for sharing through DASH is consistent with the informed consent of the participants. While obtaining IRB approvals for completed studies is typically one of the lengthiest and often rate limiting step for many submissions, ensuring compliance with human subjects protection standards is paramount. For data access, all DASH data requesters must sign a Data Use Agreement that is executed between NICHD and the requester along with their institution. The requester must agree to strict terms and conditions for data privacy and security, including prohibition on identification of subjects, data disclosure, non-distribution of data to anyone other than DASH-approved individuals and compliance with requester’s institutional information security practices. Third, if required by the study submitter, investigators requesting access to the study data must obtain approval from their institutional IRB. Fourth, the Committee that oversees data access requests for DASH verifies that the research plan submitted by data requesters are compliant with any data use limitations specified by the study submitter based on study consent. Finally, DASH is a Federal Information Security Management Act (FISMA) moderate system, ensuring that the data are encrypted at rest and in motion.

### Building the business case for centralized repositories

In recognition of the importance of demonstrating sustainability and ROI, DASH has developed an evaluation plan that includes 19 performance measures aimed at ROI, DASH value, usage and efficiency. The DASH performance evaluation plan includes measures such as the number of publications resulting from secondary use of data obtained through DASH, the impact factor of the journal these secondary studies are published in, the number of citations the original and secondary studies receive, the significances of the findings from the secondary reuse of data through DASH, timeliness of data sharing and number of new collaborations initiated from data sharing facilitated by DASH.

**Figure 1:**
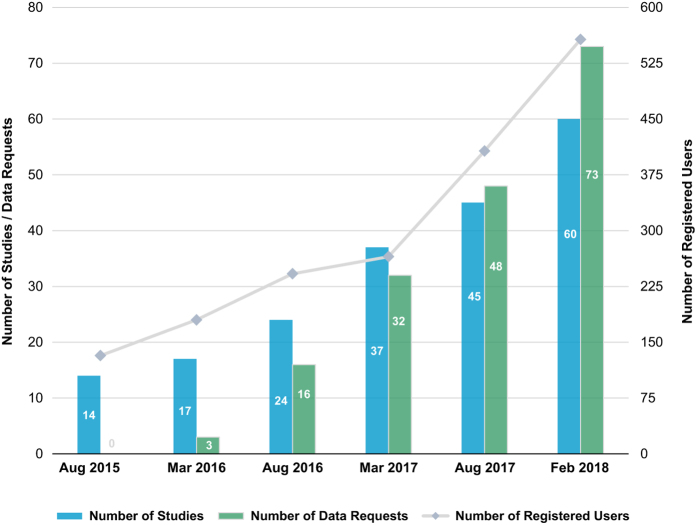
Number of Studies, Requests and Registrations in DASH (August 2015 – Feb 2018).

Although two and half years is likely insufficient to obtain an accurate assessment of the benefit-to-cost ratio of DASH or centralized data repositories more generally, the initial performance data on DASH is promising (Fig. 1). Currently there are 60 studies available through DASH – these studies span 25 clinical research study topics and have been conducted across the globe on a diverse set of study populations. The number of studies available through DASH has risen from 14 at the time of launch in August 2015 to 60 as of February 2018, averaging six studies submitted and released in DASH per quarter. There have been over 11,800 users (determined through unique Internet Protocol address) accessing DASH, with more than 550 of whom are registered users. All users can browse study information and search for data while registered users are able to submit studies, download study documentation and request data. Data access requests have consistently been processed in a timely and effective manner and the average turnaround time for data requests is 7 days. One study was requested 27 times within the first year of releasing it in DASH, highlighting again the value of DASH in data sharing.

Three manuscripts resulting from the reuse of data made available through DASH have been published^22–24^, the first one published in January 2017, less than 18 months after the launch of DASH and 12 months after data request^22^. As has been observed with other data repositories^11,25,26^, the hope is that the adoption and use of DASH will continue to grow over the next several years. Moving forward, the DASH Committee will continue to undertake an annual review of DASH using the performance evaluation plan. The performance data collected will help NICHD leadership in their assessment of the cost effectiveness of data sharing through DASH. Towards that end, DASH summarizes key lessons learned (selected ones in Table 3) on an ongoing basis and develops guidance and tools (such as the Data Preparation Tool) for investigators to incorporate as best practices for data management right from the outset and throughout the duration of the study so that they can at the end of their study ‘click to share’. The lessons learned also serves as a mechanism for the DASH implementation team to course correct if one or more of the performance evaluation metrics are not progressing as hoped.

## Discussion

DASH is one of several centralized repositories that has launched within NIH and in the private sector in the past few years to help facilitate data sharing. Many of these centralized repositories focus on data sharing from a specific topic area or sector. For example, in addition to NICHD DASH, several other NIH institutes, including the National Institute of Diabetes and Digestive and Kidney Diseases (NIDDK) and the National Heart, Lung and Blood Institute (NHLBI) have developed centralized repositories to facilitate data sharing from clinical studies funded by their institute. Other centralized repositories, including the Yale Open Data Access (YODA) Project and ClinicalStudyDataRequest.com (CSDR) are focused on sharing clinical trial data generated by private pharmaceutical and biotechnology companies. While the growth in centralized repositories is encouraging and necessary if broad data sharing is to become the new norm, care must be taken to ensure the data do not become siloed in different repositories. Efforts to facilitate the aggregation of datasets from multiple studies across repositories, such as the proposed Vivli^27^ project are important as more centralized data repositories become available. Currently such efforts are hindered by the varying level of metadata associated with studies and poor adoption of data standards. Better adoption and use of data standards will pave the way for automated tools that can help identify and aggregate datasets across multiple studies.

While centralized data repositories have an important role to play in driving and facilitating data sharing, there are still many important challenges that need to be addressed. There is a need for a greater number of tools to help facilitate data sharing among the scientific community, including tools that will aid with data provenance, lineage, and analysis. Provenance ensures that changes and harmonization that occur to that piece of data is recorded in a manner that a data user can understand. While this human-readable information does not help prove the veracity of the data, the changes would be unintelligible to the user without this information. Systems must be intelligent enough to track and verify provenance information for a wide and dynamic variety of data types that will come from increasingly varied data sources as well as a need for greater discipline among researchers in the management and documentation process^28^. Putting these systems in place will remove much of the guess-work associated with data reuse. Additional instruction for scientists so they can avail themselves of new opportunities that become possible with data sharing is another important component that needs to be augmented. There is also a need to increase the number and size of programs that exist to train informatics professionals and data curators.

Perhaps most important, though, are the policy and culture changes needed to incentivize and reward data sharing. As others have noted, while the benefits of data sharing may outweigh the costs for the community, the same does not always hold true for the individual investigator^29^. A growing number of prizes (e.g., http://asap.plos.org/) and awards for pursuing collaborative science are making data sharing a more attractive proposition for investigators but there is still a long way to go. Targeted competitions that bring the data science community together around a common challenge (e.g., the Sage Bionetworks DREAM Challenges or the SPRINT Data Analysis Challenge) are increasingly popular method for driving reuse of important datasets and a few (e.g. Data Science Bowl) have become regular, annual events. Realizing the full potential of data sharing will require changes to the incentive structure for researchers^30^. For example, data originators need to be rewarded for their contributions and should be able to include the use of their data for consideration by promotion and tenure committees. The Data Citation Index is one method through which scientists can be acknowledged and tracked on the impact of making their data available to the broader research community^31^.

In recognition of the need to broaden the acknowledgements that submitting investigators receive for their contribution, DASH requires annual progress reports from data requestors and relays this information back to the original/submitting investigator so that they are aware of the results coming from their data. Data sharing via DASH enables new collaboration among investigators not previously possible when data remained with the original study investigator. For example, sharing data in DASH in 2016 from a study that was completed in 2008 and shared only among the study investigators led to a new collaborative project among seven external investigators.

A sustained effort is needed to understand and create the policies and incentives that will be most effective in promoting data sharing and collaboration. By creating tools, infrastructure and training to make the process of data sharing simple for researchers on both the giving and receiving end of data sharing, DASH goes a long way to facilitate data sharing. With sustained support for data sharing from policy makers, the broader NIH community, non-profits, academic institutions and the publishing community, a more open and collaborative culture will continue to flourish in biomedical research.

## Methods

There were several factors that played a critically important role in the development and adoption of the DASH repository. The following sections describe the four factors that were most critical to the successful implementation of DASH: developing guiding principles, obtaining leadership support and commitment, tight coordination between technical and non-technical teams, and ongoing engagement of critical stakeholders.

### Guiding principles

Establishing and adhering to guiding principles early on was critical for the success of DASH. User-centered design was the overarching framework for building the DASH repository. Stakeholders were engaged early and often throughout the process to ensure that the repository being developed would meet their needs. It is the exact opposite of the *build and they will come* framework, which centers on the designer’s vision rather than the needs of the end user community. User-centered design is a nimbler approach to development, allowing for the development plans to adjust based on input and feedback from the end user community as the product is developed. The resulting product better meet the needs of the end user, and therefore typically experience better adoption rates.

Equally important was the adoption of design principles and technical development methods that would support a user-centered design philosophy. The key design principles included building a flexible and modular system, one that could easily evolve to meet the changing needs of end users. Another important design principle was identifying key user roles – researchers, leadership, and archive administrator – early on as a means of focusing the development efforts and obtaining feedback from the appropriate individuals along the way. Defining the core groups of end users provided a way to prioritize features based on users’ needs and feedback.

The technical development team used an agile methodology that would allow for rapid prototyping and iterative development cycles to ensure the product meets users’ needs. The DASH Committee elected to implement DASH using a cloud hosting solution for several important reasons including its cost-effectiveness, convenience and continuous availability, scalability and ability to support a diversity of devices independent of location. Adoption of a flexible, modular system will help ensure that DASH can continue to grow to meet the evolving needs of the NICHD research community.

### Leadership commitment and support

Leadership commitment and support was another critically important factor. While the need for DASH was first articulated by the scientific community during the scientific visioning process in 2012, NICHD leadership set the vision, initiated and supported the endeavor. Developing and maintaining data repositories is resource intensive. From the beginning, leadership played an important role in engaging and committing the appropriate financial and staffing resources needed to get it off the ground and ensuring their continued availability throughout the project. As the endeavor progressed, leadership continued to play a critical role in recognizing the challenges and developing solutions to address them. The DASH Committee developed guiding principles on data sharing that helped direct the technical development. Consistent leadership support has played an essential role in the development, launch and management of DASH.

### Tight coordination of technical and Non-Technical teams

A critical factor for the successful implementation and ongoing maintenance of DASH is the integration of science, policy and technology through the seamless coordination and communication between the NICHD Office of the CIO, in charge of the technical implementation and deployment, and the DASH Committee charged with overseeing the governance of its implementation. While many factors, such as the development of adoptable DASH policies through close engagement of the stakeholder community or the selection of optimal technology components such as Amazon Web Services (AWS) cloud played major roles, the coordination between the technical and non-technical teams was a crucial criterion that led to the successful deployment of DASH at NICHD. Establishing NICHD DASH as a data sharing platform at the intersection of science, policy and technology took a year and required close coordination between and among federal and contractor staff of approximately 12-14 FTEs that were composed of a diverse skillset well versed with NIH and other federal policies (human subject regulations, HIPAA and FISMA), policy and governance development for data and systems within the NIH environment, bioinformatics, data management, de-identification and curation, cloud architectural design and hosting, data and system security, and software development.

### Stakeholder engagement

Engaging stakeholders early and often was critically important for the implementation of DASH. Throughout the process, ensuring regular, ongoing communication between the policy and governance team and the technical team was essential. This ongoing dialogue helped ensure that key needs and hold-ups were discussed and addressed in a timely manner and provided an opportunity for the technical team to help the broader team understand the capabilities and limitations of the technical infrastructure. In addition to engaging a key set of internal stakeholders which included program office representatives from the division of intramural and extramural research, the DASH Committee sought insight from expert program staff of other NIH archives. As researchers themselves, program leaders were able to represent the needs and perspectives and researchers more broadly. The goal of engaging the program officers was to obtain their assessment on the need for a data repository and initiate a dialogue about the needs from the perspective of the research community they serve. Building a strategic communications approach to stakeholder engagement was critical to ensure there was support for the initiative and that the repository under development would meet the unique needs of the NICHD-funded researcher community.

With the benefit of hindsight, earlier engagement of the end users, particularly a broader group of the external researchers, would have been helpful for a more thorough usability testing of the prototype. Another benefit to earlier and more extensive engagement with the broader end user group is in more accurately estimating the level of effort involved in data curation and study documentation preparation, especially in cases where some of the DASH required documentation such as the study protocol or code book were missing. Both these challenges highlight the critical importance of engaging the NICHD-funded researchers early and often to ensure that the end product meets the needs of the user community. Based on this experience, the DASH team is in the process of preparing additional tools that help researchers prepare study data for archiving right at the outset of the study, enabling ‘click to share’ functionality.

## Additional information

**How to cite this article**: Hazra, R. *et al.* DASH, the data and specimen hub of the National Institute of Child Health and Human Development. *Sci. Data* 5:180046 doi: 10.1038/sdata.2018.45 (2018).

**Publisher’s note:** Springer Nature remains neutral with regard to jurisdictional claims in published maps and institutional affiliations.

## Figures and Tables

**Table 1:** Major DASH Goals Translated to Success Criteria.

DASH Goals	Success Criteria (Select)
Quality Data	•Cleaned, participant level data•Essential study documentations (such as study protocol, data dictionary, data collection instruments) for meaningful data use•Data collection consistent with 45 C.F.R. Part 46 and other federal clinical research regulations•Data with accurate attributions (provenance) without any errors introduced by the system (integrity), linkage to study/investigators (pedigree) and protected from loss or unauthorized access (security)
Standardization	•Standard descriptors and metadata for data and documentations•Standard templates and workflows for submissions and requests
Task Driven Access	•Query, integrate and analyze data•Answer specific research questions•Support administrative activities and reporting
Efficiency	•Efficient browsing and searching, data submissions and requests•User friendly and dynamic format and page layout•Robust workflows for data submission, access, and use•Mechanism for user feedback and adjustments for improving efficiency and performance
Compliance	•Data sharing consistent with informed consents of the participants, relying upon guidance provided by an appropriate Institutional Review Board (IRB) when the terms and conditions of data sharing are unclear or not addressed in the informed consent•Data de-identified of all 18 HIPAA identifiers•Compliance to applicable federal and NIH policies as it relates to system design, data storage, access, sharing, and use
Transparency	•Easy visibility to study portfolio and data within DASH•Study and data file descriptions for meaningful interpretation of data•‘One-stop-shop’ for NICHD supported studies stored in DASH and other archives•Linkage of NICHD funded studies stored in external archives

**Table 2:** How DASH Addressed Top Data Sharing Challenges.

Challenges	How DASH Addressed Challenges
**Data Ownership:**As the study Principal Investigator, I own the data that I generated and would like to use it for a long time	•Per NIH data sharing policies, investigators with annual funding >$500 K must share data within one year of publishing the main findings; DASH will also accept data from investigators receiving annual NIH funding of less than $500 K•Per NICHD, all NICHD funded investigators must share data in DASH or another publicly accessible repository in alignment with NIH data sharing policies•As a condition of the Data Use Agreement that all data requesters must sign to receive data access, all studies that reuse data from DASH are required to acknowledge the original study in their publication, ensuring the original study principal investigator receives credit for their contribution
**Lack of Resources:**Willing to share data but do not have the funding to prepare the data	•DASH provides guidance, tools and expertise to help investigator prepare data for sharing•NICHD has issued an R03 (small grant) funding opportunity announcement to assist investigators with archiving data (current information on the grants awarded through this funding opportunity are publicly available through the NIH Research Portfolio Online Reporting Tools (RePORT) by searching for FOA PAR-16-149)
**Informed Consent Concerns:**Willing to share data but not sure if my data can be shared	The study IRB or an equivalent Privacy Board must determine that data can be shared in DASH and is consistent with the informed consent of the participant. In some cases, the informed consent may not explicitly state broad data sharing, particularly for studies completed many years ago, before broad data sharing was feasible.
**Varying Data Types and Formats:**Do not know of any repositories that accept the various types of data I generate; Data is not in a uniform format or structure	•DASH acts as a data lake; accepting all types of clinical research data (clinical, laboratory, pathology, genomic, images, etc.) due to its flexible metadata model•Accepts data in a variety of structures and formats but is annotated with standard descriptors and metadata for easy discovery
**Data with PII:**Data contains personally identifiable information and sharing would compromise my subjects’ privacy	DASH stores only de-identified data and provides guidance and support to de-identify data of all 18 HIPAA identifiers
**Data Privacy and Security:**Concerned about data privacy and security and methods to ensure the data are used only for the agreed upon purpose	DASH has multiple safeguards in place to assure data privacy and security:•Data recipient along with their institution must execute a Data Use Agreement with NICHD for a specific research plan and agree to data privacy and recipient’s institutional information security policies, thereby holding not just the recipient but the recipient’s institution liable for any violation•Additional study-specific approvals, such as IRB approval or study-specific steering committee or PI approvals, if required by the data submitter•Data are encrypted both at rest and in transition•System is compliant with FISMA standards
**Data Readiness for Sharing:**Do not know how to prepare my data and documentation for sharing with others	DASH offers tools and guidance for researchers to prepare and annotate their data and documentation for sharing such as the Data Preparation Tool (DPT), guidance for de-identification and coding and the DASH tutorial

**Table 3:** Key Lessons Learned from DASH Implementation at NICHD.

**Data Management for Archiving**
Provide data sharing language for investigators to include in the informed consents prior to enrolling participants and initiating studies funded by NICHD
Provide guidance and tools to effectively manage data from the onset of the study and through the study life cycle so that at the end of the study, the investigator can ‘click to share’ in DASH
Provide guidance and standard templates to prepare study documentations at the outset of the study, as: •Essential study documentations varied in quality, content and structure •Significant effort is required to retroactively prepare documentations for meaningful use
Encourage use of a universal participant identifier such as Global Unique Identifier (GUID) to enhance data integration and meta-analysis
Ensure appropriate metadata standards are selected for broad searches and discovery
Coordinate with data submitters on options for de-identification of certain variables such as low frequency or rare conditions
Provide options for IRB approval for sharing data from older studies where data sharing is not explicitly stated in the informed consent or the study IRB is no longer active or for multi-site studies
**Enhancing DASH Usage and Value**
Engage investigator stakeholders during design and implementation and collect feedback from broader user community. This is critical for long term success and sustenance of DASH
Identify and engage early users to become champions and advocates of data sharing and DASH
Maintain data linkages to studies available internally in DASH and/or externally to enable data users to optimize their data search efforts
Maintain data provenance by ensuring that any data changes or edits made by data owners or authorized users is recorded by the system and kept for review and auditing purposes
Provide URL links of NICHD supported studies deposited in publicly accessible repositories other than DASH to enhance data use across NICHD supported studies
